# *catena*-Poly[[[bis­(4-ethyl­benzoato-κ^2^ *O*,*O*′)lead(II)]-μ-nicotinamide-κ^2^ *N* ^1^:*O*] monohydrate]

**DOI:** 10.1107/S1600536811026535

**Published:** 2011-07-09

**Authors:** Tuncer Hökelek, Barış Tercan, Ertan Şahin, Vedat Aktaş, Hacali Necefoğlu

**Affiliations:** a Department of Physics, Hacettepe University, 06800 Beytepe, Ankara, Turkey; b Department of Physics, Karabük University, 78050 Karabük, Turkey; c Department of Chemistry, Atatürk University, 22240 Erzurum, Turkey; d Department of Chemistry, Kafkas University, 36100 Kars, Turkey

## Abstract

In the crystal structure of the polymeric title compound, {[Pb(C_9_H_9_O_2_)_2_(C_6_H_6_N_2_O)]·H_2_O}_*n*_, the six-coordinate Pb^II^ ion is chelated by two 4-ethyl­benzoate (PEB) anions and is bridged by two nicotinamide (NA) ligands, forming a polymeric chain running along the *b* axis. The carboxyl­ate groups of the PEB ions are twisted away from the attached benzene rings by 4.0 (6) and 13.3 (5)°. The two benzene rings of the PEB ions bonded to the same metal ion are oriented at a dihedral angle of 87.4 (3)°. In the polymeric chain, the NA ligand is linked to one of the carboxyl­ate groups *via* N—H⋯O hydrogen bonding. In the crystal, adjacent polymeric chains inter­act *via* N—H⋯O and weak C—H⋯O hydrogen bonds; and the lattice water mol­ecule links with the polymeric chains *via* N—H⋯O and O—H⋯O hydrogen bonding. π–π stacking between the benzene and the pyridine rings [centroid–centroid distance = 3.805 (5) Å] and weak C—H⋯π inter­actions are also observed in the crystal structure.

## Related literature

For niacin, see: Krishnamachari (1974 ▶). For *N*,*N*-diethyl­nicotinamide, see: Bigoli *et al.* (1972 ▶). For related structures, see: Greenaway *et al.* (1984 ▶); Hökelek & Necefoğlu (1996 ▶); Hökelek *et al.* (2009*a* ▶,*b* ▶,*c* ▶,*d* ▶, 2010 ▶).
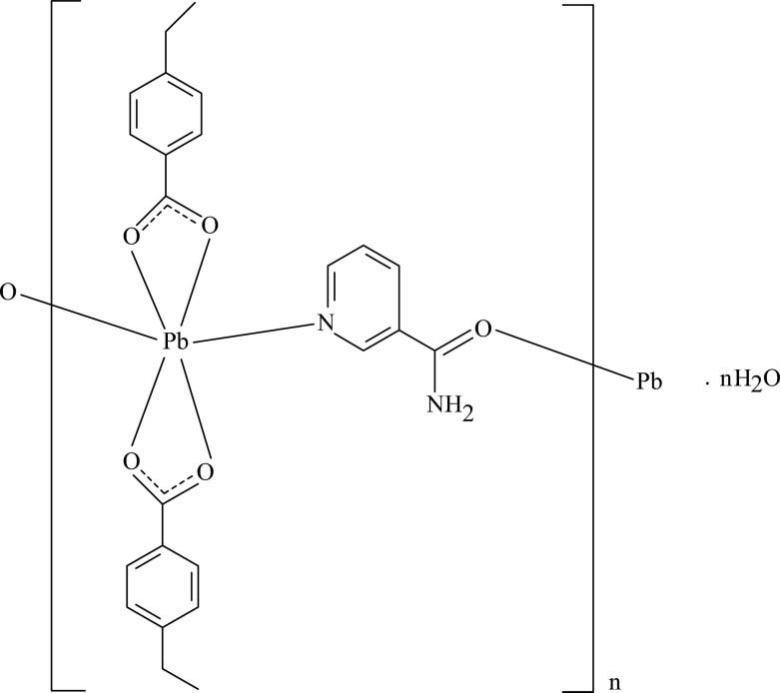


## Experimental

### 

#### Crystal data

[Pb(C_9_H_9_O_2_)_2_(C_6_H_6_N_2_O)]·H_2_O
*M* *_r_* = 645.67
Triclinic, 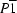

*a* = 7.8093 (2) Å
*b* = 9.7950 (3) Å
*c* = 16.9380 (5) Å
α = 90.772 (2)°
β = 91.256 (2)°
γ = 106.916 (4)°
*V* = 1239.00 (7) Å^3^
*Z* = 2
Mo *K*α radiation
μ = 6.85 mm^−1^
*T* = 294 K
0.25 × 0.20 × 0.15 mm

#### Data collection

Rigaku R-AXIS RAPID-S diffractometer
Absorption correction: multi-scan (Blessing, 1995 ▶) *T*_min_ = 0.211, *T*_max_ = 0.358
26925 measured reflections
5076 independent reflections
4208 reflections with *I* > 2σ(*I*)
*R*_int_ = 0.081

#### Refinement

*R*[*F* ^2^ > 2σ(*F* ^2^)] = 0.043
*wR*(*F* ^2^) = 0.099
*S* = 1.06
5076 reflections
314 parameters
5 restraints
H atoms treated by a mixture of independent and constrained refinement
Δρ_max_ = 1.11 e Å^−3^
Δρ_min_ = −0.96 e Å^−3^

### 

Data collection: *CrystalClear* (Rigaku/MSC, 2005 ▶); cell refinement: *CrystalClear*; data reduction: *CrystalClear*; program(s) used to solve structure: *SHELXS97* (Sheldrick, 2008 ▶); program(s) used to refine structure: *SHELXL97* (Sheldrick, 2008 ▶); molecular graphics: *Mercury* (Macrae *et al.*, 2006 ▶); software used to prepare material for publication: *WinGX* (Farrugia, 1999 ▶) and *PLATON* (Spek, 2009 ▶).

## Supplementary Material

Crystal structure: contains datablock(s) I, global. DOI: 10.1107/S1600536811026535/xu5254sup1.cif

Structure factors: contains datablock(s) I. DOI: 10.1107/S1600536811026535/xu5254Isup2.hkl

Additional supplementary materials:  crystallographic information; 3D view; checkCIF report

## Figures and Tables

**Table 1:** Selected bond lengths (Å)

Pb1—O1	2.423 (5)
Pb1—O2	2.670 (5)
Pb1—O3	2.356 (5)
Pb1—O4	2.638 (5)
Pb1—O5	2.537 (5)
Pb1—N1	2.787 (5)

**Table 2:** Hydrogen-bond geometry (Å, °) *Cg* is the centroid of the C11–C16 ring.

*D*—H⋯*A*	*D*—H	H⋯*A*	*D*⋯*A*	*D*—H⋯*A*
N2—H2*A*⋯O1	0.74 (17)	2.15 (16)	2.834 (8)	155 (19)
N2—H2*B*⋯O6	0.82 (16)	2.06 (15)	2.871 (9)	172 (15)
O6—H61⋯O2^i^	0.89 (5)	2.01 (7)	2.811 (8)	149 (7)
O6—H62⋯O3^ii^	0.90 (9)	1.93 (9)	2.822 (9)	169 (10)
C13—H13⋯O4^iii^	0.93	2.47	3.313 (11)	150
C19—H19⋯O6^iv^	0.93	2.43	3.323 (10)	162
C4—H4⋯*Cg*^v^	0.93	2.87	3.712 (8)	151

Symmetry codes: (i) 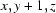; (ii) 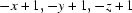; (iii) 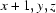; (iv) 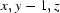; (v) 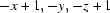.

**Fig. 1.**
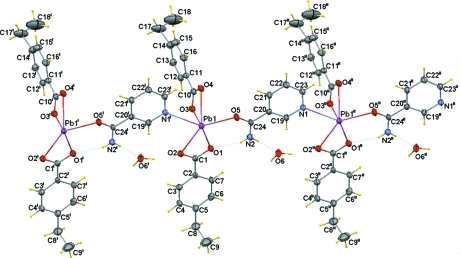
The molecular structure of the title compound with the atom-numbering scheme. Displacement ellipsoids are drawn at the 50% probability level. Primed atoms are generated by the symmetry operators: (') x, 2 - y, z, ('') x, 1 - y, z. Dashed lines indicate the N—H···O hydrogen-bondings.

**Fig. 2.**
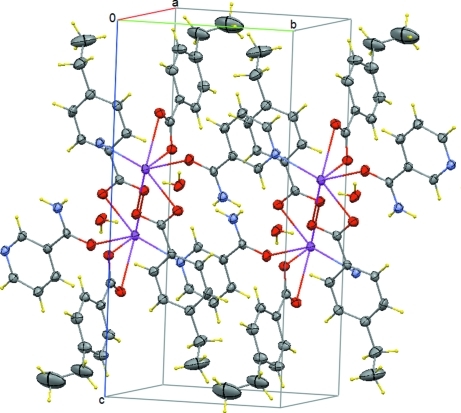
The crystal structure of the title complex.

## supplementary crystallographic information

### Comment

As a part of our ongoing investigation on transition metal complexes of nicotinamide (NA), one form of niacin (Krishnamachari, 1974), and/or the nicotinic acid derivative *N*,*N*-diethylnicotinamide (DENA), an important respiratory stimulant (Bigoli *et al.*, 1972), the title compound was synthesized and its crystal structure is reported herein.

In the crystal structure of the title compound, each Pb^II^ ion is coordinated by two 4-ethylbenzoate (PEB) and one nicotinamide (NA) ligands (Fig. 1), while symmetry related NA ligands bridge the Pb^II^ ions forming polymeric chains along the *b* axis (Fig. 2). The two PEB ions act as bidentate ligands, while the NA is monodentate ligand (Fig. 1). The crystal structures of similar complexes of Cd^II^, Co^II^, Mn^II^, Zn^II^ and Pb^II^ ions, [Cd(C_8_H_5_O_3_)_2_(C_6_H_6_N_2_O)_2_(H_2_O)].H_2_O, (II) (Hökelek *et al.*, 2009*a*), [Co(C_9_H_10_NO_2_)_2_(C_6_H_6_N_2_O)(H_2_O)_2_], (III) (Hökelek *et al.*, 2009*b*), [Mn(C_9_H_10_NO_2_)_2_(C_6_H_6_N_2_O)(H_2_O)_2_], (IV) (Hökelek *et al.*, 2009*c*), [Zn_2_(DENA)_2_(C_7_H_5_O_3_)_4_].2H_2_O, (V) (Hökelek & Necefoğlu, 1996), [Zn(C_8_H_8_NO_2_)_2_(C_6_H_6_N_2_O)_2_].H_2_O, (VI) (Hökelek *et al.*, 2009*d*) and [Pb(C_8_H_7_O_2_)_2_(C_6_H_6_N_2_O)]_n_, (VII) (Hökelek *et al.*, 2010), have also been reported. In (II) and (VII), the two benzoate ions are coordinated to the Cd and Pb atoms, respectively, as bidentate ligands. In the other structures one of the benzoate ligands acts as a bidentate ligand, while the other is monodentate ligand.

The average Pb—O bond length (Table 1) is 2.525 (5) Å and the Pb1 atom is displaced out of the least-squares planes of the carboxylate groups (O1/C1/O2) and (O3/C10/O4) by 0.1782 (3) Å and 0.3574 (3) Å, respectively. The O1/C1/O2 and O3/C9/O4 carboxylate planes form dihedral angles of 4.01 (63)° and 13.32 (53)°, respectively, with benzene rings A(C2-C7) and B(C11-C16), while the angles between rings A, B and C (N1/C19-C23) are A/B = 87.36 (26), A/C = 8.95 (22) and B/C = 78.76 (20)°. One of the intramolecular N-H···O hydrogen bonds (Table 2) links the NA ligand to the uncoordinated water molecule, while the other N-H···O hydrogen bond links the NA ligand to one of the carboxylate groups of the PEB ions acting as a bidentate ligand. In (I), the O1-Pb1-O2 and O3-Pb1-O4 angles are 51.10 (15)° and 51.95 (16)°, respectively. The corresponding O-M-O (where M is a metal) angles are 52.91 (4)° and 53.96 (4)° in (II), 60.70 (4)° in (III), 58.45 (9)° in (IV), 58.3 (3)° in (V), 60.03 (6)° in (VI), 51.09 (6)° and 51.71 (5)° in (VII) and 55.2 (1)° in [Cu(Asp)_2_(py)_2_] (where Asp is acetylsalicylate and py is pyridine) [(VIII); Greenaway *et al.*, 1984].

In the crystal structure, N—H···O, O—H···O and C—H···O hydrogen bonds (Table 2) link adjacent chains into a two-dimensional network parallel to the *bc* plane. The π···π contact between the benzene and pyridine rings, Cg1—Cg3^i^, [symmetry code: (i) -x, -y, 1 - z, where Cg1 and Cg3 are the centroids of the rings A(C2-C7) and C (N1/C19-C23), respectively, may further stabilize the structure, with centroid-centroid distance of 3.805 (5) Å. There also exists a weak C—H···π interaction involving the benzene ring B(C11-C16) (Table 2).

### Experimental

The title compound was prepared by the reaction of Pb(NO_3_)_2_ (1.656 g, 5 mmol) in H_2_O (100 ml) and nicotinamide (1.220 g, 10 mmol) in H_2_O (50 ml) with sodium 4-ethylbenzoate (1.720 g, 10 mmol) in H_2_O (100 ml). The mixture was filtered and set aside to crystallize at ambient temperature for three weeks, giving colorless single crystals.

### Refinement

Atoms H2A and H2B (for NH_2_) and H61 and H62 (for H_2_O) were located in a difference Fourier map and refined isotropically. The C-bound H-atoms were positioned geometrically with C—H = 0.93, 0.97 and 0.96 Å, for aromatic, methylene and methyl H-atoms, respectively, and constrained to ride on their parent atoms, with *U*_iso_(H) = k × *U*_eq_(C), where k = 1.5 for methyl H-atoms and k = 1.2 for all other H-atoms. The highest peak and deepest hole are located 0.94 and 0.90 Å, respectively, from Pb1.

### Crystal data

**Table:**

[Pb(C_9_H_9_O_2_)_2_(C_6_H_6_N_2_O)]·H_2_O	*Z* = 2
*M**_r_* = 645.67	*F*(000) = 628
Triclinic, *P*1	*D*_x_ = 1.731 Mg m^−^^3^
Hall symbol: -P 1	Mo *K*α radiation, λ = 0.71073 Å
*a* = 7.8093 (2) Å	Cell parameters from 6051 reflections
*b* = 9.7950 (3) Å	θ = 2.2–26.4°
*c* = 16.9380 (5) Å	µ = 6.85 mm^−^^1^
α = 90.772 (2)°	*T* = 294 K
β = 91.256 (2)°	Block, colorless
γ = 106.916 (4)°	0.25 × 0.20 × 0.15 mm
*V* = 1239.00 (7) Å^3^	

### Data collection

**Table:**

Rigaku R-AXIS RAPID-S diffractometer	5076 independent reflections
Radiation source: fine-focus sealed tube	4208 reflections with *I* > 2σ(*I*)
graphite	*R*_int_ = 0.081
ω scans	θ_max_ = 26.4°, θ_min_ = 2.2°
Absorption correction: multi-scan (Blessing, 1995)	*h* = −9→9
*T*_min_ = 0.211, *T*_max_ = 0.358	*k* = −12→10
26925 measured reflections	*l* = −21→21

### Refinement

**Table:**

Refinement on *F*^2^	Primary atom site location: structure-invariant direct methods
Least-squares matrix: full	Secondary atom site location: difference Fourier map
*R*[*F*^2^ > 2σ(*F*^2^)] = 0.043	Hydrogen site location: inferred from neighbouring sites
*wR*(*F*^2^) = 0.099	H atoms treated by a mixture of independent and constrained refinement
*S* = 1.06	*w* = 1/[σ^2^(*F*_o_^2^) + (0.0277*P*)^2^ + 1.8361*P*] where *P* = (*F*_o_^2^ + 2*F*_c_^2^)/3
5076 reflections	(Δ/σ)_max_ < 0.001
314 parameters	Δρ_max_ = 1.11 e Å^−^^3^
5 restraints	Δρ_min_ = −0.96 e Å^−^^3^

### Special details

**Table:**

Geometry. All esds (except the esd in the dihedral angle between two l.s. planes) are estimated using the full covariance matrix. The cell esds are taken into account individually in the estimation of esds in distances, angles and torsion angles; correlations between esds in cell parameters are only used when they are defined by crystal symmetry. An approximate (isotropic) treatment of cell esds is used for estimating esds involving l.s. planes.
Refinement. Refinement of F^2^ against ALL reflections. The weighted R-factor wR and goodness of fit S are based on F^2^, conventional R-factors R are based on F, with F set to zero for negative F^2^. The threshold expression of F^2^ > 2sigma(F^2^) is used only for calculating R-factors(gt) etc. and is not relevant to the choice of reflections for refinement. R-factors based on F^2^ are statistically about twice as large as those based on F, and R- factors based on ALL data will be even larger.

### Fractional atomic coordinates and isotropic or equivalent isotropic displacement parameters (Å^2^)

**Table:**

	*x*	*y*	*z*	*U*_iso_*/*U*_eq_	
Pb1	0.08609 (4)	0.15890 (3)	0.394120 (15)	0.05863 (12)	
O1	0.2512 (8)	0.2967 (5)	0.5057 (3)	0.0772 (15)	
O2	0.2289 (7)	0.0686 (5)	0.5205 (3)	0.0700 (13)	
O3	0.3813 (6)	0.2084 (5)	0.3496 (3)	0.0666 (12)	
O4	0.1966 (7)	0.1944 (5)	0.2477 (3)	0.0664 (12)	
O5	0.0783 (8)	0.4092 (5)	0.3616 (3)	0.0789 (15)	
O6	0.3512 (9)	0.8292 (6)	0.5453 (4)	0.0839 (17)	
H61	0.347 (11)	0.919 (4)	0.550 (5)	0.09 (3)*	
H62	0.425 (11)	0.808 (11)	0.582 (5)	0.14 (4)*	
N1	0.0879 (8)	−0.1057 (6)	0.3322 (3)	0.0626 (15)	
N2	0.2113 (10)	0.5633 (7)	0.4600 (4)	0.0671 (17)	
H2A	0.24 (2)	0.509 (17)	0.482 (10)	0.211*	
H2B	0.26 (2)	0.640 (16)	0.482 (9)	0.211*	
C1	0.2776 (9)	0.1960 (8)	0.5468 (4)	0.0588 (17)	
C2	0.3632 (9)	0.2337 (7)	0.6261 (4)	0.0572 (16)	
C3	0.3884 (10)	0.1280 (8)	0.6761 (4)	0.0676 (19)	
H3	0.3562	0.0336	0.6581	0.081*	
C4	0.4593 (11)	0.1614 (8)	0.7507 (4)	0.072 (2)	
H4	0.4733	0.0893	0.7832	0.087*	
C5	0.5101 (11)	0.2995 (9)	0.7782 (5)	0.075 (2)	
C6	0.4914 (12)	0.4041 (9)	0.7300 (5)	0.086 (3)	
H6	0.5287	0.4983	0.7483	0.103*	
C7	0.4179 (10)	0.3741 (7)	0.6538 (4)	0.0678 (19)	
H7	0.4055	0.4473	0.6219	0.081*	
C8	0.5923 (15)	0.3367 (12)	0.8615 (6)	0.114 (4)	
H8A	0.6335	0.2584	0.8804	0.137*	
H8B	0.6954	0.4205	0.8593	0.137*	
C9	0.465 (2)	0.364 (2)	0.9167 (8)	0.226 (10)	
H9A	0.5276	0.4072	0.9644	0.339*	
H9B	0.3777	0.2761	0.9287	0.339*	
H9C	0.4062	0.4280	0.8934	0.339*	
C10	0.3483 (10)	0.2031 (7)	0.2747 (4)	0.0613 (17)	
C11	0.4949 (9)	0.2061 (7)	0.2208 (4)	0.0578 (16)	
C12	0.6732 (10)	0.2396 (8)	0.2472 (4)	0.0664 (19)	
H12	0.7031	0.2632	0.3001	0.080*	
C13	0.8050 (11)	0.2375 (9)	0.1947 (5)	0.077 (2)	
H13	0.9236	0.2624	0.2128	0.092*	
C14	0.7663 (13)	0.1999 (12)	0.1170 (6)	0.094 (3)	
C15	0.5895 (13)	0.1637 (11)	0.0916 (5)	0.095 (3)	
H15	0.5603	0.1349	0.0392	0.114*	
C16	0.4540 (11)	0.1691 (9)	0.1423 (5)	0.078 (2)	
H16	0.3363	0.1479	0.1234	0.094*	
C17	0.9025 (19)	0.166 (2)	0.0606 (8)	0.193 (9)	
H17A	0.9823	0.1221	0.0880	0.289*	
H17B	0.8430	0.1051	0.0166	0.289*	
C18	0.997 (3)	0.309 (3)	0.0350 (13)	0.328 (18)	
H18A	1.0379	0.3035	−0.0177	0.491*	
H18B	1.0975	0.3498	0.0700	0.491*	
H18C	0.9172	0.3673	0.0356	0.491*	
C19	0.1215 (9)	−0.2116 (7)	0.3749 (4)	0.0593 (17)	
H19	0.1658	−0.1909	0.4264	0.071*	
C20	0.0926 (9)	0.6504 (7)	0.3452 (4)	0.0573 (16)	
C21	0.0247 (11)	0.6222 (8)	0.2695 (4)	0.071 (2)	
H21	0.0012	0.5307	0.2478	0.086*	
C22	−0.0087 (12)	−0.2704 (8)	0.2257 (5)	0.079 (2)	
H22	−0.0530	−0.2880	0.1740	0.094*	
C23	0.0241 (10)	−0.1386 (8)	0.2593 (4)	0.0654 (19)	
H23	0.0003	−0.0671	0.2293	0.079*	
C24	0.1279 (9)	0.5335 (7)	0.3905 (4)	0.0567 (16)	

### Atomic displacement parameters (Å^2^)

**Table:**

	*U*^11^	*U*^22^	*U*^33^	*U*^12^	*U*^13^	*U*^23^
Pb1	0.0721 (2)	0.04518 (17)	0.05984 (18)	0.01901 (13)	−0.00048 (12)	0.00349 (11)
O1	0.113 (4)	0.062 (3)	0.062 (3)	0.034 (3)	−0.010 (3)	0.005 (2)
O2	0.093 (4)	0.055 (3)	0.063 (3)	0.025 (3)	−0.010 (3)	−0.001 (2)
O3	0.069 (3)	0.068 (3)	0.063 (3)	0.020 (3)	−0.006 (2)	0.003 (2)
O4	0.065 (3)	0.069 (3)	0.068 (3)	0.024 (2)	−0.005 (2)	0.011 (2)
O5	0.113 (4)	0.040 (3)	0.083 (4)	0.023 (3)	−0.015 (3)	0.003 (2)
O6	0.110 (5)	0.062 (4)	0.088 (4)	0.043 (3)	−0.043 (3)	−0.020 (3)
N1	0.081 (4)	0.046 (3)	0.062 (4)	0.022 (3)	−0.012 (3)	−0.001 (3)
N2	0.080 (4)	0.054 (4)	0.067 (4)	0.019 (3)	−0.006 (3)	0.000 (3)
C1	0.065 (4)	0.053 (4)	0.058 (4)	0.017 (3)	0.001 (3)	0.006 (3)
C2	0.074 (5)	0.044 (4)	0.056 (4)	0.020 (3)	0.007 (3)	0.009 (3)
C3	0.079 (5)	0.065 (5)	0.061 (4)	0.025 (4)	−0.006 (4)	−0.003 (4)
C4	0.099 (6)	0.059 (5)	0.065 (5)	0.032 (4)	−0.010 (4)	0.011 (4)
C5	0.095 (6)	0.063 (5)	0.070 (5)	0.030 (4)	−0.018 (4)	−0.004 (4)
C6	0.106 (7)	0.053 (5)	0.090 (6)	0.012 (4)	−0.004 (5)	−0.018 (4)
C7	0.092 (6)	0.044 (4)	0.066 (5)	0.019 (4)	−0.007 (4)	0.007 (3)
C8	0.139 (10)	0.119 (9)	0.085 (7)	0.042 (7)	−0.032 (6)	−0.025 (6)
C9	0.30 (2)	0.36 (3)	0.091 (10)	0.21 (2)	−0.035 (12)	−0.051 (13)
C10	0.067 (5)	0.052 (4)	0.064 (5)	0.016 (3)	−0.006 (4)	−0.001 (3)
C11	0.064 (4)	0.056 (4)	0.053 (4)	0.018 (3)	−0.007 (3)	−0.002 (3)
C12	0.074 (5)	0.064 (5)	0.063 (4)	0.022 (4)	−0.005 (4)	0.001 (3)
C13	0.062 (5)	0.094 (6)	0.079 (6)	0.031 (4)	−0.002 (4)	0.000 (4)
C14	0.079 (6)	0.126 (8)	0.086 (6)	0.042 (6)	0.008 (5)	0.000 (6)
C15	0.088 (7)	0.148 (9)	0.057 (5)	0.046 (6)	−0.003 (4)	−0.004 (5)
C16	0.069 (5)	0.099 (6)	0.070 (5)	0.030 (5)	−0.009 (4)	0.009 (4)
C17	0.106 (11)	0.36 (3)	0.114 (11)	0.072 (14)	0.043 (8)	0.046 (13)
C18	0.21 (3)	0.60 (6)	0.19 (2)	0.14 (3)	0.084 (18)	0.05 (3)
C19	0.066 (4)	0.047 (4)	0.066 (4)	0.020 (3)	−0.007 (3)	−0.006 (3)
C20	0.062 (4)	0.048 (4)	0.062 (4)	0.016 (3)	0.005 (3)	−0.001 (3)
C21	0.101 (6)	0.048 (4)	0.067 (5)	0.025 (4)	−0.012 (4)	−0.007 (3)
C22	0.111 (7)	0.060 (5)	0.063 (5)	0.024 (4)	−0.022 (4)	−0.004 (4)
C23	0.078 (5)	0.056 (4)	0.065 (5)	0.025 (4)	−0.008 (4)	0.008 (3)
C24	0.070 (5)	0.034 (4)	0.065 (4)	0.014 (3)	0.000 (3)	0.010 (3)

### Geometric parameters (Å, °)

**Table:**

Pb1—O1	2.423 (5)	C9—H9A	0.9600
Pb1—O2	2.670 (5)	C9—H9B	0.9600
Pb1—O3	2.356 (5)	C9—H9C	0.9600
Pb1—O4	2.638 (5)	C11—C10	1.474 (10)
Pb1—O5	2.537 (5)	C11—C12	1.397 (10)
Pb1—N1	2.787 (5)	C11—C16	1.379 (10)
Pb1—C1	2.924 (7)	C12—C13	1.378 (10)
Pb1—C10	2.856 (8)	C12—H12	0.9300
O1—C1	1.277 (8)	C13—C14	1.365 (12)
O2—C1	1.266 (8)	C13—H13	0.9300
O3—C10	1.286 (8)	C14—C15	1.379 (12)
O4—C10	1.240 (8)	C15—H15	0.9300
O5—C24	1.254 (8)	C16—C15	1.390 (11)
O6—H61	0.89 (2)	C16—H16	0.9300
O6—H62	0.90 (2)	C17—C18	1.454 (17)
N1—C19	1.356 (8)	C17—C14	1.547 (12)
N1—C23	1.320 (8)	C17—H17A	0.9700
N2—H2A	0.72 (15)	C17—H17B	0.9700
N2—H2B	0.82 (15)	C18—H18A	0.9600
C1—C2	1.479 (9)	C18—H18B	0.9600
C2—C3	1.400 (9)	C18—H18C	0.9600
C2—C7	1.388 (9)	C19—C20^i^	1.389 (9)
C3—C4	1.366 (10)	C19—H19	0.9300
C3—H3	0.9300	C20—C21	1.369 (10)
C4—C5	1.367 (10)	C20—C19^ii^	1.389 (9)
C4—H4	0.9300	C21—C22^ii^	1.377 (10)
C5—C6	1.358 (11)	C21—H21	0.9300
C5—C8	1.530 (11)	C22—C21^i^	1.377 (10)
C6—H6	0.9300	C22—H22	0.9300
C7—C6	1.393 (11)	C23—C22	1.356 (10)
C7—H7	0.9300	C23—H23	0.9300
C8—H8A	0.9700	C24—N2	1.320 (9)
C8—H8B	0.9700	C24—C20	1.474 (9)
C9—C8	1.458 (13)		
			
O1—Pb1—O2	51.10 (15)	C5—C8—H8B	109.2
O1—Pb1—O4	123.27 (17)	C9—C8—C5	112.2 (10)
O1—Pb1—O5	78.18 (17)	C9—C8—H8A	109.2
O1—Pb1—N1	130.47 (17)	C9—C8—H8B	109.2
O1—Pb1—C1	25.49 (17)	H8A—C8—H8B	107.9
O1—Pb1—C10	103.1 (2)	C8—C9—H9A	109.5
O2—Pb1—N1	82.33 (16)	C8—C9—H9B	109.5
O2—Pb1—C1	25.64 (16)	C8—C9—H9C	109.5
O2—Pb1—C10	106.17 (19)	H9A—C9—H9B	109.5
O3—Pb1—O1	79.48 (18)	H9A—C9—H9C	109.5
O3—Pb1—O2	82.21 (16)	H9B—C9—H9C	109.5
O3—Pb1—O4	51.95 (16)	O3—C10—Pb1	54.6 (4)
O3—Pb1—O5	91.12 (18)	O3—C10—C11	118.7 (6)
O3—Pb1—N1	78.26 (17)	O4—C10—Pb1	67.3 (4)
O3—Pb1—C1	80.81 (18)	O4—C10—O3	121.3 (7)
O3—Pb1—C10	26.41 (18)	O4—C10—C11	120.0 (7)
O4—Pb1—O2	130.39 (15)	C11—C10—Pb1	169.6 (5)
O4—Pb1—N1	71.75 (16)	C12—C11—C10	122.1 (6)
O4—Pb1—C1	132.48 (17)	C16—C11—C10	119.0 (7)
O4—Pb1—C10	25.69 (17)	C16—C11—C12	118.8 (7)
O5—Pb1—O2	129.25 (16)	C11—C12—H12	120.0
O5—Pb1—O4	75.76 (16)	C13—C12—C11	119.9 (7)
O5—Pb1—N1	145.37 (17)	C13—C12—H12	120.0
O5—Pb1—C1	103.61 (18)	C12—C13—H13	119.1
O5—Pb1—C10	84.56 (19)	C14—C13—C12	121.9 (8)
N1—Pb1—C1	106.98 (18)	C14—C13—H13	119.1
N1—Pb1—C10	71.28 (19)	C13—C14—C15	118.0 (8)
C10—Pb1—C1	107.2 (2)	C13—C14—C17	123.2 (10)
C1—O1—Pb1	99.8 (4)	C15—C14—C17	117.5 (10)
C1—O2—Pb1	88.4 (4)	C14—C15—C16	121.6 (8)
C10—O3—Pb1	99.0 (4)	C14—C15—H15	119.2
C10—O4—Pb1	87.0 (4)	C16—C15—H15	119.2
C24—O5—Pb1	140.0 (5)	C11—C16—C15	119.7 (8)
H61—O6—H62	113 (9)	C11—C16—H16	120.2
C19—N1—Pb1	124.7 (4)	C15—C16—H16	120.2
C23—N1—Pb1	117.3 (4)	C14—C17—H17A	111.6
C23—N1—C19	117.0 (6)	C14—C17—H17B	111.6
C24—N2—H2A	121 (10)	C18—C17—C14	100.7 (15)
C24—N2—H2B	131 (10)	C18—C17—H17A	111.6
H2B—N2—H2A	107 (10)	C18—C17—H17B	111.6
O1—C1—Pb1	54.8 (3)	H17A—C17—H17B	109.4
O1—C1—C2	117.6 (6)	C17—C18—H18A	109.5
O2—C1—Pb1	65.9 (4)	C17—C18—H18B	109.5
O2—C1—O1	120.6 (6)	C17—C18—H18C	109.5
O2—C1—C2	121.8 (6)	H18A—C18—H18B	109.5
C2—C1—Pb1	171.1 (5)	H18A—C18—H18C	109.5
C3—C2—C1	120.9 (6)	H18B—C18—H18C	109.5
C7—C2—C1	121.2 (6)	N1—C19—C20^i^	123.0 (6)
C7—C2—C3	117.9 (6)	N1—C19—H19	118.5
C2—C3—H3	119.4	C20^i^—C19—H19	118.5
C4—C3—C2	121.1 (7)	C19^ii^—C20—C24	124.0 (6)
C4—C3—H3	119.4	C21—C20—C19^ii^	117.3 (6)
C3—C4—C5	120.7 (7)	C21—C20—C24	118.6 (6)
C3—C4—H4	119.7	C20—C21—C22^ii^	119.9 (7)
C5—C4—H4	119.7	C20—C21—H21	120.1
C4—C5—C8	120.5 (8)	C22^ii^—C21—H21	120.1
C6—C5—C4	119.2 (7)	C21^i^—C22—H22	120.6
C6—C5—C8	120.3 (8)	C23—C22—C21^i^	118.8 (7)
C5—C6—C7	121.8 (7)	C23—C22—H22	120.6
C5—C6—H6	119.1	N1—C23—C22	123.9 (7)
C7—C6—H6	119.1	N1—C23—H23	118.1
C2—C7—C6	119.3 (7)	C22—C23—H23	118.1
C2—C7—H7	120.4	O5—C24—N2	122.2 (6)
C6—C7—H7	120.4	O5—C24—C20	119.0 (6)
C5—C8—H8A	109.2	N2—C24—C20	118.9 (6)
			
O2—Pb1—O1—C1	−2.2 (4)	O4—Pb1—C10—C11	−136 (3)
O3—Pb1—O1—C1	−90.7 (5)	O5—Pb1—C10—O3	103.7 (4)
O4—Pb1—O1—C1	−120.0 (4)	O5—Pb1—C10—O4	−68.2 (4)
O5—Pb1—O1—C1	175.9 (5)	O5—Pb1—C10—C11	156 (3)
N1—Pb1—O1—C1	−26.2 (5)	N1—Pb1—C10—O3	−101.5 (4)
C10—Pb1—O1—C1	−102.6 (5)	N1—Pb1—C10—O4	86.7 (4)
O1—Pb1—O2—C1	2.2 (4)	N1—Pb1—C10—C11	−49 (3)
O3—Pb1—O2—C1	85.0 (4)	C1—Pb1—C10—O3	1.1 (4)
O4—Pb1—O2—C1	106.1 (4)	C1—Pb1—C10—O4	−170.8 (4)
O5—Pb1—O2—C1	−0.1 (5)	C1—Pb1—C10—C11	53 (3)
N1—Pb1—O2—C1	164.1 (4)	Pb1—O1—C1—O2	4.3 (8)
C10—Pb1—O2—C1	96.2 (4)	Pb1—O1—C1—C2	−174.6 (5)
O1—Pb1—O3—C10	−153.1 (4)	Pb1—O2—C1—O1	−3.8 (7)
O2—Pb1—O3—C10	155.1 (4)	Pb1—O2—C1—C2	175.0 (6)
O4—Pb1—O3—C10	−4.5 (4)	Pb1—O3—C10—O4	8.8 (7)
O5—Pb1—O3—C10	−75.4 (4)	Pb1—O3—C10—C11	−170.6 (5)
N1—Pb1—O3—C10	71.4 (4)	Pb1—O4—C10—O3	−7.8 (6)
C1—Pb1—O3—C10	−179.0 (4)	Pb1—O4—C10—C11	171.6 (6)
O1—Pb1—O4—C10	42.4 (5)	Pb1—O5—C24—N2	−1.0 (13)
O2—Pb1—O4—C10	−22.3 (5)	Pb1—O5—C24—C20	178.7 (5)
O3—Pb1—O4—C10	4.6 (4)	Pb1—N1—C19—C20^i^	169.0 (5)
O5—Pb1—O4—C10	107.6 (4)	C23—N1—C19—C20^i^	0.4 (11)
N1—Pb1—O4—C10	−84.6 (4)	Pb1—N1—C23—C22	−169.6 (6)
C1—Pb1—O4—C10	12.0 (5)	C19—N1—C23—C22	−0.1 (12)
O1—Pb1—O5—C24	−2.5 (8)	O1—C1—C2—C3	176.8 (7)
O2—Pb1—O5—C24	−0.7 (9)	O1—C1—C2—C7	−1.9 (11)
O3—Pb1—O5—C24	−81.5 (8)	O2—C1—C2—C3	−2.1 (11)
O4—Pb1—O5—C24	−131.7 (8)	O2—C1—C2—C7	179.2 (7)
N1—Pb1—O5—C24	−152.3 (7)	C1—C2—C3—C4	−176.8 (7)
C1—Pb1—O5—C24	−0.7 (8)	C7—C2—C3—C4	2.0 (11)
C10—Pb1—O5—C24	−107.2 (8)	C1—C2—C7—C6	177.4 (7)
O1—Pb1—N1—C19	29.5 (7)	C3—C2—C7—C6	−1.3 (11)
O1—Pb1—N1—C23	−161.8 (5)	C2—C3—C4—C5	−0.9 (13)
O2—Pb1—N1—C19	10.9 (5)	C3—C4—C5—C6	−0.9 (14)
O2—Pb1—N1—C23	179.6 (6)	C3—C4—C5—C8	−179.0 (9)
O3—Pb1—N1—C19	94.5 (6)	C4—C5—C6—C7	1.6 (14)
O3—Pb1—N1—C23	−96.8 (5)	C8—C5—C6—C7	179.7 (9)
O4—Pb1—N1—C19	148.1 (6)	C4—C5—C8—C9	−103.5 (14)
O4—Pb1—N1—C23	−43.3 (5)	C6—C5—C8—C9	78.5 (15)
O5—Pb1—N1—C19	169.1 (5)	C2—C7—C6—C5	−0.5 (13)
O5—Pb1—N1—C23	−22.2 (7)	C12—C11—C10—Pb1	−59 (3)
C1—Pb1—N1—C19	18.1 (6)	C12—C11—C10—O3	−11.5 (10)
C1—Pb1—N1—C23	−173.3 (5)	C12—C11—C10—O4	169.0 (7)
C10—Pb1—N1—C19	121.0 (6)	C16—C11—C10—Pb1	119 (3)
C10—Pb1—N1—C23	−70.4 (5)	C16—C11—C10—O3	165.8 (7)
O1—Pb1—C1—O2	−176.0 (7)	C16—C11—C10—O4	−13.6 (10)
O2—Pb1—C1—O1	176.0 (7)	C16—C11—C12—C13	1.1 (11)
O3—Pb1—C1—O1	84.8 (5)	C10—C11—C12—C13	178.4 (7)
O3—Pb1—C1—O2	−91.1 (4)	C12—C11—C16—C15	1.0 (12)
O4—Pb1—C1—O1	78.9 (5)	C10—C11—C16—C15	−176.4 (8)
O4—Pb1—C1—O2	−97.0 (4)	C11—C12—C13—C14	−1.6 (13)
O5—Pb1—C1—O1	−4.1 (5)	C12—C13—C14—C15	0.1 (15)
O5—Pb1—C1—O2	179.9 (4)	C12—C13—C14—C17	−167.0 (11)
N1—Pb1—C1—O1	159.5 (4)	C13—C14—C15—C16	2.0 (16)
N1—Pb1—C1—O2	−16.5 (4)	C17—C14—C15—C16	169.9 (11)
C10—Pb1—C1—O1	84.3 (5)	C11—C16—C15—C14	−2.6 (15)
C10—Pb1—C1—O2	−91.6 (4)	C18—C17—C14—C13	−85.6 (17)
O1—Pb1—C10—O3	27.2 (4)	C18—C17—C14—C15	107.2 (17)
O1—Pb1—C10—O4	−144.7 (4)	C19^ii^—C20—C21—C22^ii^	1.3 (12)
O1—Pb1—C10—C11	79 (3)	C24—C20—C21—C22^ii^	−179.7 (7)
O2—Pb1—C10—O3	−25.7 (4)	N1—C23—C22—C21^i^	0.4 (13)
O2—Pb1—C10—O4	162.5 (4)	O5—C24—C20—C19^ii^	171.9 (7)
O2—Pb1—C10—C11	26 (3)	O5—C24—C20—C21	−7.1 (10)
O3—Pb1—C10—O4	−171.8 (7)	N2—C24—C20—C19^ii^	−8.4 (11)
O3—Pb1—C10—C11	52 (3)	N2—C24—C20—C21	172.7 (7)
O4—Pb1—C10—O3	171.8 (7)		

Symmetry codes: (i) *x*, *y*−1, *z*; (ii) *x*, *y*+1, *z*.

### Hydrogen-bond geometry (Å, °)

**Table:**

Cg is the centroid of the C11–C16 ring.

**Table:**

*D*—H···*A*	*D*—H	H···*A*	*D*···*A*	*D*—H···*A*
N2—H2A···O1	0.74 (17)	2.15 (16)	2.834 (8)	155 (19)
N2—H2B···O6	0.82 (16)	2.06 (15)	2.871 (9)	172 (15)
O6—H61···O2^ii^	0.89 (5)	2.01 (7)	2.811 (8)	149 (7)
O6—H62···O3^iii^	0.90 (9)	1.93 (9)	2.822 (9)	169 (10)
C13—H13···O4^iv^	0.93	2.47	3.313 (11)	150
C19—H19···O6^i^	0.93	2.43	3.323 (10)	162
C4—H4···Cg^v^	0.93	2.87	3.712 (8)	151

Symmetry codes: (ii) *x*, *y*+1, *z*; (iii) −*x*+1, −*y*+1, −*z*+1; (iv) *x*+1, *y*, *z*; (i) *x*, *y*−1, *z*; (v) −*x*+1, −*y*, −*z*+1.
